# Supplementary figures and images for: The relationship between voting restrictions and COVID-19 case and mortality rates between US counties
Source: PLoS One. 2022 Jun 1;17(6):e0267738. doi: 10.1371/journal.pone.0267738 (PMC9159582; doi:10.1371/journal.pone.0267738)

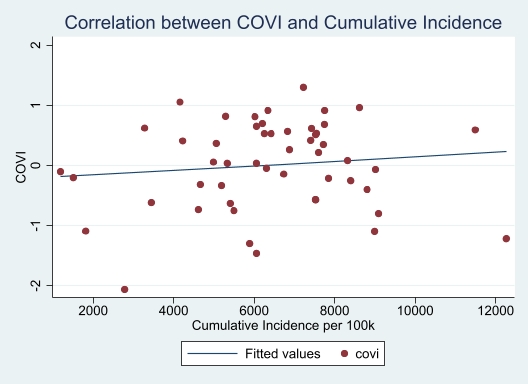


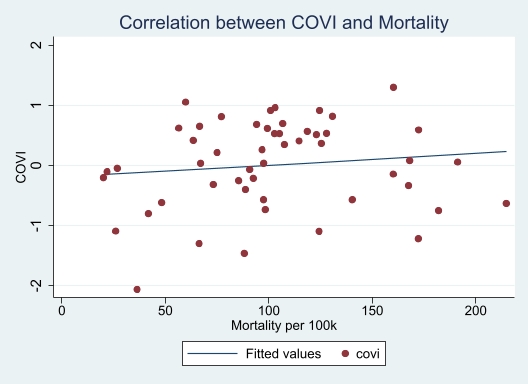


Appendix-1: The correlation between COVI and Cumulative Incidence rate and mortality rate.

Supplement: S1 Appendix — (DOCX) [file pone.0267738.s001.docx]
