# Supplementary material for: The relationship between voting restrictions and COVID-19 case and mortality rates between US counties
Source: PLoS One. 2022 Jun 1;17(6):e0267738. doi: 10.1371/journal.pone.0267738 (PMC9159582; doi:10.1371/journal.pone.0267738)
Supplement: Attachment — Submitted filename: responsetoreviewersRR1Submitted.docx [file pone.0267738.s002.docx]

Emily Chenette, PhD

Editor-in-Chief

Plos One

March 2, 2022

Dear Dr. Emily Chenette,

Thank you for your correspondence regarding our manuscript entitled “The Relationship Between Voting Restrictions and COVID-19 Transmission and Mortality Rates within US Counties” in *Plos One*.

In an appended document, we have outlined in detail the changes made to the manuscript and have highlighted new text in bold font both in the response and as a tracked change within the body text of the manuscript itself. We address each comment point-by-point. We found the comments of the reviewers to be very helpful and feel that the manuscript has been improved as a result of their advice.

We hope you find these revisions acceptable, as we have sought to be responsive to the comments we received. Thank you again for your consideration.

Yours sincerely,

Corresponding Author

Associate Professor in Epidemiology

School of Public Health

University of Alberta

E-mail: [pabayo@ualberta.ca](mailto:pabayo@ualberta.ca)

Phone: 780-492-9954

Fax: 780-492-0364

Response to Reviewers

Reviewer #1: Review of “The Relationship Between Voting Restrictions and COVID-19 Transmission and Mortality Rates within US Counties”

1. The title contains two misconceptions: 1. “transmission” refers to spread of the virus in time but this is a cross-sectional study of identified cases after one year. 2. the study is not of differences “within counties”. It examines differences among counties.

Response:

We thank the reviewer for this comment. We have changed the title of the manuscript to:

**The Relationship Between Voting Restrictions and COVID-19 Case and Mortality Rates	between US Counties**

2. First sentence: “Participating in voting is related to beneficial health outcomes at the community level [1, 2]”. The references say that fewer people vote in communities where people have more health problems. That does not mean that voting has an effect on health status. People with impaired health may vote less or both could be a result of other factors. It is doubtful that people vote on the basis of concerns for preventive public health. The exception may have been in the 2020 election because of Trump’s lies re: COVID19. The turnout for the 2020 elections was very high by historical standards and probably cost Trump the election (<https://link.springer.com/article/10.1007/s00148-020-00820-3>). The mortality rate in a county was related to percent Trump voters and testing, corrected for numerous factors associated with interpersonal spread of a highly contagious virus, but that is likely the result of Trump voters’ devotion to Trump’s lies rather than voter suppression which is primarily a state level, not county level, policy. (<https://rdcu.be/cCwdm>). This study controls for only a few of the potential confounders.

Response:

We thank the reviewer for this comment. The sentence was changed to the following:

**The right to vote offers marginalized communities the opportunity to participate in the political process, thereby enjoying a policy environment that better represents their needs. This potentially leads to beneficial population health outcomes (1-3). For example, voters who believe in access to Medicaid but confront barriers to access to voting may not have their voices heard in the electoral process, and therefore not have access to health services.**

We also added the following in the limitations section of our discussion:

**Participating in voting is associated with beneficial health outcomes at the county level (1-3). However, this relationship likely suffers from endogeneity. Thus, it is likely that while voting may lead to improved health outcomes, better health outcomes may also be associated with increased voter participation.**

Also, since partisanship at the county level may be correlated with COVI, we have included proportion in a county that voted for Donald Trump in the 2016 election. However, this did not substantively change our results.

3. The report repeats the prevalent cliché among many people who study social factors and health, “social determinants of health”. Social and economic factors contribute to increase or decreased risk of certain injuries and illnesses but none are determinative. Also, the effects vary in opposite directions depending on the outcome studied. Social support is related to positive health outcomes for some chronic health problems but social contacts in a pandemic spread the disease.
   Voter suppression is a threat to democracy and may result in candidates adverse to public health winning elections but the study reported in this paper does not adequately explicate the issue.

Response:

These are very good points. In response, we have re-written large portions of the paper.

There is substantial evidence that the social determinants of health are bi-directional in nature. However, the bulk of randomized controlled trials on social policy experiments suggest that social policies enacted by politicians do produce impacts on health, and small improvements in socio-economic circumstances lead to measurable changes in health measures. (Courtin, E., S. Kim, S. Song, W. Yu, and P. Muennig. 2020. Can Social Policies Improve Health? A Systematic Review and Meta-Analysis of 38 Randomized Trials. Milbank Quarterly 98: 297-371. Since that paper, was published, an additional RCT showed that changes in health are exquisitely sensitive to changes in EITC benefits.)

We also agree that strong social support networks can have unknown impacts on contagion. They can mean that the hub is exposed to more people or they can mean that the hub is receiving cautious messages to take precautions. We have removed any language that may be seen as speculative.

The present study is one initial study in which we try to understand an understudied association. We hope that it will to further studies using causal designs to more fully understand the association between voting restrictions and health outcomes. By doing so we can gain knowledge on the health impact of current political trends.

Reviewer #2: Thank you for the opportunity to review this manuscript.

Overall, I think the authors make an important contribution to scholarship at the nexus between public health and political science. The authors' argument is as novel as it is plausible. The theory section does a good job of establishing a link between anti-democratic reforms and public health outcomes while doing a good job of citing the relevant political science literature. The methodological approach strikes me as pragmatic. In an ideal world, a county-level indicator for cost of voting would have been preferable to the state-level measure that was used here. Yet, the authors did the best that they could with the data that was available to them. Multilevel models tend to be overused; here, it was more than appropriate to opt for such models. All of this translates into an original, nifty contribution that will be well cited. Thus, my recommendation is that this paper be published with the following (minor) revisions.

4. My first suggestion relates to how partisanship is addressed in the methods section. The reason the authors are controlling for states' partisan leanings is that the relationship of interest could be driven by this third factor. I suggest that the authors make this clearer in two ways. First, for consistency, they should remove any mention of "ideology," as the concept they are interested in is rather "partisanship." Second, I would be more explicit about why partisanship is a crucial control to have: this variable influences *both* voting restrictions and COVID-19 rates. In other words, partisanship is a confounder that meets the back-door criterion.

Response:

We thank the reviewers for this comment, we have changed "ideology" to "partisanship." We have also added the following:

State-level political partisanship is a potential confounder in the association between voting restrictions and COVID cases **because it can be associated with both voting restrictions and COVID-19 outcomes**. **For example,** **local** governments and **national** political parties sometimes politicized public health responses to the pandemic.

5. A second suggestion would be to make the figures more intelligible. Figures 1a and 1b lack axis labels, and the titles are not at all clear. It was initially hard to know what I was looking at. What is the horizontal axis, and why does it go from 1 to 5? What are the vertical axes, and why are they different in each figure? I would like to see a detailed legend explaining all of this very clearly. Relatedly, why are there to confidence intervals surrounding the point estimates? This would help readers assess visually whether the plotted heterogeneous relationships meet standard levels of statistical significance. Finally, what is the total number of cases (i.e., unique observations)? I cannot find this information at the bottom of either regression table. Is it 3142 observations (one per county), or are there repeated county observations?

Response:

We thank the reviewers for this suggestion. We have included the axis labels and titles. We have also corrected the horizontal axis to be from -2 to +2. We have also added the number of the counties in the titles of each figure.

6. A third suggestion would be to present (or at least mention in the text) some additional *bivariate* descriptive findings. Which states are the best and worst in terms of voting, and how do these states rank in terms of COVID-19 cases and deaths? Are some of the hardest-hit counties in states figuring among the worst culprits? These are only suggestions. I am confident the authors will be able to include one or two such data snippets to illustrate their paper's main argument.

Response:

We have included bivariate associations between COVI and state-level COVID-19 outcomes. The following was added in the statistical analysis section:

**We first determined the bivariate associations between COVI and state-level COVID-19 case and mortality rates. Scatter plots were also created to visualize these bivariate relationships.**

And the following was added to the results section:

**The bivariate associations between COVI and COVID-19 outcomes indicate that a one SD increase in COVI was associated with an increase in state-level COVID-19 case (ß=256.8, 95% CI=-376.7, 890.3) and mortality rates (ß=5.6, 95%CI=-7.4,18.7), but these estimates were not statistically significant. The scatterplots of the association between COVI and COVID-19 cumulative incidence rate and mortality rate can be found in Appendix-1.**

7. To me, the paper's main weakness is that it uses a state-level indicator for the explanatory variable whereas the dependent variable is measured at the county level. As an additional assurance that the results are robust despite the different levels of observation, I would like to see reported correlation coefficients between COVI and each of the two COVID-19 outcomes *at the state level*. Put differently, I would like the authors to aggregate their county-level COVID-19 indicators to see if there is a bivariate association between the explanatory variable and each dependent variable at the state level. The authors might also find it worthwhile to include scatterplots showing the raw, bivariate relationship between their explanatory and dependent variables.

Response:

Thank you for this important comment. We have included the bivariate associations as indicated to our response to number 5. Also, we have included scatterplots showing the raw bivariate relationship between COVI and the COVID-19 outcomes in the appendix.

8. Additionally, the paragraph in page 4 starting by "A final contributor to geographical variation is..." should be revised for clarity, as the main substantive idea was not easy to follow. One sentence, in particular, seems to bundle together COVID-19 and voter restrictions in explaining the election of politicians who are averse to public health. I do not understand this point. Are the authors arguing that COVID-19 cases and deaths depress support for those politicians who are more likely to support measures to stop the spread of the virus? I am not convinced that COVID-19 exposure changed how people voted (see Mendoza Avina & Sevi, 2021). If anything, COVID-19 shifted the electorate towards politicians more willing to embrace sound public health measures (i.e., Democrats; see Warshaw, Vavreck, & Baxter-King, 2020).

Response:

We thank the reviewer for this comment. We would like to clarify that our paper does not examine whether COVID changed the way the public voted (reverse causality of our exposure and outcome). We are examining whether voting barriers, which limits the political power of socioeconomic groups most affected by COVID, affect the overall COVID risk. We made extensive changes to the paragraph (as well as other parts of the paper).

A final contributor to geographical variation is funding for the social determinants of health. Anti-poverty policies, such as Medicaid expansion and financial aid, are designed to reduce material hardship and improve the health of socio-economically-disadvantaged populations. Poverty is associated with a larger burden of disease than smoking and obesity combined [9]. Yet voter restrictions, **which have a disproportionate effect on individuals from lower socioeconomic backgrounds and people of color,** may produce a shift from candidates who are more likely to support policies that address such socioeconomic inequities compared to those who are less likely to support them. People from such socio-demographic groups are also more likely to work in essential services and businesses that are at higher risk for COVID-19 transmission since they cannot work from home and are more likely to be in workplaces where social distancing is not possible [10, 11,^-^12]. **These essential workers would also be disproportionately affected by restrictive voting laws, which may lead to elected officials who discount public health measures.**

9. Finally, I do not know whether the authors did this intentionally or not, but I noticed that they tend to avoid using the terms "Democratic" and "Republican" to describe those politicians who tend to support or oppose certain policies. For example, they argue that nonwhite voters and those from socioeconomic disadvantaged backgrounds are more likely to support politicians who support public health measures to slow the spread of the virus. This is technically true, but a simpler and more accurate characterization of the political dynamics at hand is that minority and poor voters support the Democratic Party, which fully supports the policies in question (unlike the Republican Party). The most influential theories of vote choice and preference formation tell us that most voters will support or oppose candidates primarily because of the party to which they belong, not the policies they promote. Voters follow their preferred party's issue positions, meaning that most of them would vote the same way regardless of their respective party's public health platform. Thus I would encourage the authors to characterize citizens' electoral behavior in terms of partisanship rather than public policy.

Response:

We appreciate this comment. We wish to avoid political bias or generalizations, and would prefer using non-specific language. We focus on the underlying issues rather than the political party, in part because several Republicans (e.g., Mitt Romney) support Democratic policies in this arena. Likewise, a meaningful number of low income minority voters voted Republican. We appreciate the reviewer’s point about party platforms, but respectfully decline to incorporate this change.

Reviewer #3: The authors test whether voting restrictions are associated with COVID-19 transmission and death rates, and whether the nature of this association differs as a function of (1) the proportion of Black residents, and (2) median household income.

There is a lot to like about this work. This paper poses a (to my knowledge) novel and important question, and tests it in a relatively compelling fashion. The introduction is well crafted and concise, and makes a logical case for the proposed associations.

10. My primary concern is that the authors may not have adequately controlled for the possible confounding influence of Republicanism. That is - as the authors note - the pandemic was highly politicized, with Republican politicians tending to impose fewer restrictions. At the same time, voting restrictions (e.g., voter ID laws) are also more common in Republican-leaning areas. Thus, it is possible that any observed associations between voting restrictions and covid transmission / death rates are due to greater Republicanism, rather than the voting restrictions per se.

    The authors attempt to address this potential confound by controlling (i.e., statistically adjusting for) state-level Republicanism. And, in fact, adding this control variable to the model looking at transmission rates does in fact "knock out" their proposed effects, yielding an association between voting restrictions and transmission rates that is no longer statistically significant.

    Importantly, the authors DO still find an association between voting restrictions and death rates, even after adjusting for state-level Republicanism. However, this state-level measure is much less fine-grained than the county-level measures that constitute their primary IV (county-level voting restrictions; specifically, the Cost of Voting Index) and DVs (covid transmission and death rates). As a result, there is greater imprecision in their control variable, which could potentially lead to a kind of "type 2" error here -- that is, a failure to detect a real confounding influence of area Republicanism. Fortunately, there is an easy fix for this: use a county-level index of area Republicanism. The easiest option would be to simply look at Republican-versus-Democratic voting at the county level (e.g., for the previous two elections, akin to the Cook Partisan Voting Index). This information is readily available -- e.g., here: <https://electionlab.mit.edu/data>.

    If the primary effects of interest hold up when controlling for this more fine-grained measure of Republicanism, I (and, I think, readers) would find the results more compelling.

Response:

We thank the reviewer for this suggestion. We have re-conducted the analyses by removing the CVPI while adding % Trump voters in the 2016 election at the county-level. Similar estimates were obtained. We have added the new table of results.

Also, we made the following changes to the methods and results sections where we have incorporated proportion Trump voters within US counties, rather than the CPVI.

Please see our extensive changes to the Methods and Results sections throughout.

11. A couple of other smaller comments and concerns:

    In addition to the need for a county-level measure of Republicanism (which I really think is critical), I had a second concern about the authors' measure of Republicanism: I think that the Cook partisan voting index isn't ideal, insofar as it measures an area's degree of Republicanism relative to the national average in the two previous elections. Given the two-party, "us versus them" nature of American politics, though--and the way that COVID was politicized specifically along party lines--I don't think that it's RELATIVE Republicanism that is of interest, but simply a more direct measure of an area's absolute position on the Republican-Democratic spectrum. (Statistically, things will probably look very similar, but conceptually I think that this measure will make more sense to readers.)

Response:

We have mostly re-written the methods and results sections and made substantive changes throughout the paper to address these concerns. We also re-ran all of our analyses.

12. I would also like to see what happens to the interaction effects (i.e., the interaction between voting restrictions and (1) proportion of Black residents and (2) median income) when the authors also control for the INTERACTION between voting restrictions and county-level Republicanism. This is the more stringent test of their proposed interaction, and I think that it's warranted given possible associations between county level Republicanism and both median income and racial diversity.

Response:

We thank the reviewer for this suggestion. As suggested, we have included a COVI and county-level Proportion Trump voters interaction term in our analyses, when including the COVI-Proportion Black and COVI-Median income interaction terms. The findings did not change. But we do note this throughout. We apologize for not pointing to specific changes, as the changes we made were broad.

13. As a psychologist by training, I also want more insight into the mechanism behind these associations. In other words, WHY do voting restrictions relate to covid transmission and death rates? The authors speculate as to a few potential mechanisms, but do not actually test any. Of course, I recognize that this is an inherent limitation of the datasets they used, but I would suggest that the authors acknowledge this limitation a bit more clearly, and perhaps also suggest some alternative possible explanations for this association (in addition to their preferred/proposed explanation).

Response:

This is another very important point. In addition to the extensive changes to the discussion, please see, in particular, changes to the limitations section within the discussion.
